# INTERNATIONAL STANDARDS FOR NONDESTRUCTIVE TESTING A Report on the Sixth Plenary Meeting of ISO TC 135, Yokohama, Japan, May 11–15, 1987

**DOI:** 10.6028/jres.092.040

**Published:** 1987-12-01

**Authors:** Leonard Mordfin

**Affiliations:** U.S. Delegate to ISO/TC 135, Office of Nondestructive Evaluation, National Bureau of Standards, Gaithersburg, MD 20899

It is almost axiomatic that international standards facilitate trade. The sale of products by one country to another is certainly made easier when the buyer country knows that the goods it is purchasing conform to established standards; standards which both the buyer and seller countries may well have helped to formulate. It is for just this reason that the U.S. Department of Commerce strongly advocates the acceptance of international technical standards based on U.S. technology. The effects of international standards on testing services may not be as obvious as they are on products but they are just as significant.

Although the producer in a seller country and the user in a buyer country may both be cognizant of the international standards for a given product, there is often disagreement on the means for verifying the conformance of the product to the standards. Clearly, the producer must test the product before it leaves his plant in order to be sure that it satisfies the standards (or any other provisions that were made part of the contract). The buyer, however, may be unwilling to accept the seller’s assurances. He may opt to have the product retested in his own country. He may charge the costs of these tests to the manufacturer; but whether or not he does this, these costs raise the total cost of the product in the buyer country. Situations such as this make it difficult for a foreign producer to compete against a domestic producer for a share of the market in any given country, aside from any import tariffs that might be imposed by the buyer country.

In an effort to promote the sale of American products abroad, the Office of the U.S. Trade Representative has been active in international negotiations to alleviate this situation. Most of the proposals seek agreements whereby test data generated in the seller country would be accepted in the buyer country, provided that the tests were carried out in an accredited laboratory. The conditions for accreditation are still to be formulated, but it is quite clear that they will have to be contingent upon mutual agreements regarding the test methods and the qualifications of the individuals who perform the tests. In short, international standards for test methods and personnel qualifications are a prerequisite to international acceptance of test data.

For many products, tests to evaluate quality are destructive. That is, the quality of the product is evaluated, at least in part, by sampling tests of a mechanical or chemical nature which destroy the usefulness of the samples. There are numerous products, however, for which sampling and destructive testing are inappropriate and for which the quality of each and every item must be verified nondestructively. Such products are either too expensive and unique to be destroyed, or else they impact directly upon public safety. Examples include airplanes, nuclear reactors, refineries, metals processing equipment and steam generators. International agreements regarding the acceptance of test data on such products must necessarily await international standards for nondestructive test methods and for the certification of nondestructive testing personnel.

The development of such standards is the responsibility of Technical Committee 135 on Non-Destructive Testing in the International Organization for Standardization (ISO/TC 135). (The ISO continues to use the hyphenated spelling rather than the now widely accepted version, nondestructive testing or, simply, NDT.)

The technology of NDT is generally newer than that of mechanical testing, and the development of standards for NDT, accordingly, is not as far advanced as it is for mechanical testing. Although there is a large variety of NDT methods, seven are in comparatively wide use: ultrasonics, x-ray radiography, eddy currents, magnetic particles, liquid penetrants, visual examination and leak testing. Two other methods, acoustic emission testing and neutron radiography, might be classed as “rapidly emerging.”

TC 135 is organized into subcommittees (SCs) along similar lines. SC 2 on Surface Methods deals with liquid penetrant and magnetic particle testing. SC 3 on Acoustic Methods is responsible for ultrasonic and acoustic emission testing. SC 4 on Electromagnetic Methods is chiefly concerned with eddy current testing. SC 5 on Radiation Methods is responsible for x-ray, gamma ray, and neutron radiography, and SC 6 is on Leak Detection Methods. SC 7 was established only a few years ago to develop an international standard for the qualification and certification of NDT personnel for all of the methods.

At the invitation of the host country, the Sixth Plenary Meeting of ISO/TC 135 was held in Yokohama, Japan, during the week of May 11–15, 1987. Meetings of SC 2 and SC 3 were also scheduled during the same time frame, as was a meeting of Working Group 1 (WG 1) of SC 3, which is concerned with ultrasonic reference blocks.

Eight countries were represented at the meetings: The U.S.S.R., which serves as the secretariat for both TC 135 and SC 2; the U.S., which is the secretariat for SC 3; Italy, whose delegate to the meetings is the convenor for SC 3/WG 1; plus Canada, France, Federal Republic of Germany, Republic of Korea and, of course, Japan. The U.S. delegation of six members was exceeded in size only by the Japanese delegation; the Soviet delegation had four persons.

The U.S. delegation was headed by Dr. Leonard Mordfin of the National Bureau of Standards, who chairs the U.S. Technical Advisory Group for ISO/TC 135 (“the TAG”). (This Group is responsible for assessing the consensus U.S. position on matters being developed or balloted in the Technical Committee.) Other members of the U.S. delegation included Dr. Donald G. Eitzen of NBS, who was chairman-elect of SC 3, plus four representatives from the private sector: W. E. Lawrie, the U.S. member of SC 3/WG 1; J. D. Marble, the secretary of SC 3; C. W. McKee, vice chairman of the U.S. Technical Advisory Group; and Dr. M. C. Tsao, who is the principal developer of a working document on the characterization of search units and sound fields for ultrasonic nondestructive testing. This document is based upon ASTM standards. One of the U.S. delegation’s principal objectives at the meeting was to advance this document toward international acceptance. (This objective is representative of the TAG’s overall goal, which is the development and promulgation of international standards for NOT which are consistent with the best practices of American industry.)

The U.S. delegation had, in fact, several objectives which it had set for itself prior to the ISO meetings. As things turned out, all of these objectives were attained. There were two occurrences, both unexpected, which may have contributed to this successful record. The first was the absence of a delegate from the United Kingdom. The U.K.’s position in recent years has run somewhat counter to the main thrust of ISO/TC 135 activities, and this has tended to cause delays and to limit progress. The second occurrence was even more surprising and it took place, or at least it began, prior to the meeting itself.

About two hours before the scheduled opening of the plenary meeting on May 11, a member of the host Japanese delegation notified Drs. Mordfin and Eitzen that the Soviet delegation wished to meet with them immediately. The Americans assented. At the meeting, the spokesman for the Soviet delegation announced that the chairman of TC 135, Professor V. V. Kljuev, had resigned from this position because” … he was no longer working in NDT” and had not come to Yokohama. Professor Kljuev is an eminent Soviet scientist with considerable expertise in NOT, so this development was quite unexpected. Even more unexpected was the Soviet spokesman’s next remark, which was a request that Dr. Mordfin chair the week’s meetings of TC 135. For a secretariat to appoint someone other than one of its own countrymen to chair an ISO committee meeting may well be unprecedented. To the other delegates at the meeting it was certainly unheard of. Needless to say, Dr. Mordfin accepted the invitation. Although the chair is a nonpartisan position, Dr. Mordfin’s appointment unquestionably eased the U.S. delegation’s attainment of its objectives at the meetings.

In retrospect, the Soviet move was a wise one. The Soviet nominee to replace Professor Kljuev as permanent chairman of TC 135 is Dr. Y. K. Fedosenko. His qualifications for the position are impeccable but his English is poor … and TC 135 meetings are conducted in English. Hence Dr. Fedosenko’s appointment was delayed until the end of the TC 135 meetings at which time he pledged to improve his English before the next TC 135 meeting in 1989.

Aside from this, it is a matter of record that the positions of the U.S. and the U.S.S.R. in ISO TC 135 have been generally similar and quite compatible over the last few years. In fact, in matters under debate in TC 135, the U.S. finds itself allied with the Soviet Union more often than with, for example, the U.K. or France. The parallel objectives of the U.S. and the U.S.S.R. were quite evident in the Yokohama meetings. It is apparent, therefore, that the Soviet appointment of Mordfin benefited both countries.

Other notable appointments during the meeting were Mr. A. P. Degtjarev as the new permanent chairman of SC 2 on Surface Methods and Dr. Eitzen as the new permanent chairman of SC 3 on Acoustic Methods. Both men are highly acclaimed in their fields. However, Mr. Degtjarev, like Dr. Fedosenko, is new to TC 135 whereas Dr. Eitzen has been a member of the U.S. delegation to the last three plenary meetings. With this background Dr. Eitzen has, in fact, been fulfilling the chairman’s responsibilities for SC 3 since September 1986, when the previous chairman resigned. Under his deft leadership in Yokohama, the subcommittee reviewed international comments which had been submitted on the U.S. working document on the characterization of ultrasonic search units and sound fields, and a resolution was adopted elevating the next revision of the document to formal ISO Draft Proposal status.

During the year prior to the Yokohama meetings, seven proposals for new work items were letter balloted through TC 135 for addition to the committee’s Program of Work. All were accepted. Five of the seven proposals had been submitted by the U.S. One of these proposals was for the development of a standard for ultrasonic reference blocks; this was the basis for the establishment of Working Group 1 in SC 3. Another proposal was for a secondary calibration method for acoustic emission transducers; it is the U.S. intent that this be compatible with the acoustic emission measurement system developed at NBS. The other three new work items proposed by the U.S. deal with the standardization of neutron radiography. A team of U.S. experts in this field is already preparing drafts for ISO consideration, based on ASTM standards. In an effort to expedite this activity, the U.S. delegation sought and obtained a resolution directing the German secretariat of SC 5 on Radiation Methods to poll its members regarding the establishment of a new working group on neutron radiography with an American convenor.

The U.S. delegation was also successful in securing a resolution relating to personnel qualification and certification. An ISO Draft Proposal on this topic is at considerable variance with American practices. Hence, the U.S. delegation sought to invite the Canadian secretariat of SC 7 on Personnel Qualification to hold its next meeting in the United States. The American Society for Nondestructive Testing, which has offered to host this meeting, feels that the two approaches to NDT personnel qualification could, perhaps, be harmonized more effectively in an American venue.

It is not at all uncommon at technical meetings for significant accomplishments to be recorded outside of the meeting rooms. This was true in Yokohama as well. Informal discussions between members of the U.S. delegation and Mr. E. Julliard of the French delegation led to a tentative agreement for the exchange of draft nondestructive testing standards which are under development in the two countries. The proposed agreement, which would involve AFNOR standards in France and ASTM and ANSI standards in the United States, is expected to facilitate the standards development process by reducing duplication of effort and by drawing the two countries into closer alignment in TC 135. Detailed procedures for implementing the agreement remain to be worked out but preliminary exchanges have already been effected. These involved French and English vocabularies for liquid penetrant testing and ultrasonic testing as a first step toward standard ISO vocabularies for these two nondestructive test methods.

Many working drafts dealing with nondestructive test methods were furthered during the week of meetings in Yokohama, most of them with significant U.S. input. It is not practical to describe this progress here in detail, but some of the drafts which were advanced in this way include documents dealing with procedures and equipment for magnetic particle testing, vocabulary for liquid penetrant inspection, ultrasonic reference blocks, ultrasonic inspection of forgings, and x-ray radiography of castings. It was also resolved that the U.S. will submit proposals for new work items to develop ISO vocabularies on magnetic particle testing and acoustic emission testing. In addition, procedures for updating three existing ISO standards, dealing with surface methods of nondestructive testing, were reaffirmed.

Subject to approval by the German member body of ISO, the next meeting of TC 135 will be held in Berlin in 1989. Meetings that may be arranged in the meantime include SC 4 on Electromagnetic Methods in Bulgaria; SC 7 on Personnel Qualification in the United States; working group on radiography of castings in Germany; and working group on neutron radiography in Japan.

All of the delegates were uniformly complimentary about the outstanding arrangements which had been made by the Japanese hosts for these meetings. The International Conference Center in Yokohama is an exceptional facility for meetings of this kind, the banquet at the grand Hotel New Grand was exquisite, and the city of Yokohama itself proved to be thoroughly delightful.

